# Orchestrating a Heist: Uptake and Storage of Metals by Apicomplexan Parasites

**DOI:** 10.1099/mic.0.001114

**Published:** 2021-12-13

**Authors:** Megan A. Sloan, Dana Aghabi, Clare R. Harding

**Affiliations:** Wellcome Centre for Integrative Parasitology, Institute for Infection, Immunity and Inflammation, University of Glasgow, UK

## Introduction

The acquisition and storage of metals has been a preoccupation of life for millennia. Transition metals, in particular iron, copper, and zinc, have vital roles within cells. However, metals also make dangerous cargos; inappropriate uptake or storage of transition metals leads to cell death. This paradox has led to cells developing elegant and frequently redundant mechanisms for fine-tuning local metal concentrations. In the context of infection, pathogens must overcome further hurdles, as hosts act to weaponize metal availability to prevent pathogen colonisation and spread.

The Apicomplexa are a broad family of obligate intracellular eukaryotic parasites infecting a range of hosts from marine invertebrates to mammals. They share key metabolic and structural features, as well as complex life cycles with phases of sexual and asexual replication, which frequently occur in different hosts (Harding and Frischknecht, 2020; Jacot et al., 2016; Striepen et al., 2007). The best studied Apicomplexa are; *Plasmodium* spp., cause of malaria, one of the most deadly infectious diseases; *Toxoplasma gondii*, a ubiquitous pathogen of warm blooded animals which causes miscarriage and blindness; and *Cryptosporidium* spp., a leading cause of diarrheal mortality in children under 5. Here, we summarise the work that has been done on iron, zinc and copper in the context of apicomplexan parasites, focusing on the transporters required. Each metal presents distinct challenges to the parasite, e.g. labile iron is a source of dangerous reactive oxygen species and free copper is almost non-existent within mammalian hosts, however by summarising what is known about metal transport in these organisms, we hope to provide a basis for further study of this fascinating topic.

## Iron ingress and imprisonment

Iron is an essential nutrient for the vast majority of known organisms where it plays a crucial role in core processes including oxidative phosphorylation and DNA replication and repair. Iron is primarily utilized by cells as part of either heme (Kloehn et al., 2020), iron sulphur (Fe-S) clusters (Dellibovi-Ragheb et al., 2013; LaGier et al., 2003) or diiron group cofactors (Yamasaki et al., 2021).

The importance of iron to the growth of apicomplexan parasites during mammalian infection is well known. Iron supplementation in mice was shown to increase *P*. *yoelii* burden in the liver (Goma et al., 1996), while iron deficiency may be associated with reduced risk of malaria, though the choice of markers used (e.g. ferritin saturation) impacts risk estimates (Muriuki et al., 2019). Additionally treatment with iron chelators has been shown to suppress parasite growth *in vitro* and *in vivo* (Ferrer et al., 2012; Pollack et al., 1987; Thipubon et al., 2015). However, a mechanism for this is not well understood, as the therapeutic effects appear variable with infection stage and host as well as the chelator and its mode of administration (Bunnag et al., 1992; Ferrer et al., 2012; Gordeuk et al., 1992; Portugal et al., 2011; Thuma et al., 1998). *Plasmodium* spp. also require iron in their mosquito host, and iron accumulation in the mosquito has been linked to infection susceptibility (Maya-Maldonado et al., 2021). In *T*. *gondii*, iron has been shown to be important in parasite replication and pathogenesis *in vitro* and i*n vivo* (Almeida et al., 2019; Dimier and Bout, 1998; Mahmoud, 1999; Oliveira et al., 2020) although there have been no clinical studies. There has also been little work on the importance of iron to *Cryptosporidium*, although anaemia was not associated with *Cryptosporidium* prevalence in one trial (Mengist et al., 2015). Due to the lack of the respiratory chain in *Cryptosporidium*, it is likely that the parasite’s iron requirements are lower than other Apicomplexa, although iron is likely still required by the parasite (Kloehn et al., 2020; LaGier et al., 2003; Miller et al., 2018).

Acquisition of iron is non-trivial. In biological systems iron is readily oxidised to the ferric form which is poorly soluble at physiological pH and therefore not readily available for uptake. As obligate intracellular pathogens, any iron must be subverted from the host, however a conserved host defence is to limit available iron, known as nutritional immunity. These interactions are important as iron availability is often a key determinant of infection outcome (Clark et al., 2014; Dimier and Bout, 1998; Oliveira et al., 2020). There are two main options for the Apicomplexa to acquire host iron, the parasites could take up and recycle host iron-containing proteins, or they could access the cytosolic labile iron pool (LIP) directly. Despite its abundance in its erythrocyte hosts, *Plasmodium* do not appear to access iron from haemoglobin, or other host-heme containing proteins, as they lack heme oxygenase (Sigala et al., 2012). Instead, *Plasmodium* uses haemoglobin catabolism as a source of amino acids (Liu et al., 2006), and the majority of host heme is crystallised into hemozoin. *T*. *gondii* also ingests host cell material during infection (Dou et al., 2014), however the digestion of iron-containing proteins has not been confirmed and the source of parasite iron remains opaque.

The host cell LIP makes an attractive source for parasite iron as it is likely that pores, formed in the parasitophorous vacuolar membrane by parasite proteins (Garten et al., 2018; Gold et al., 2015), could permit iron from the LIP to enter the parasitophorous vacuole. From the intravacuolar space, iron could then be moved into the parasite by specific transporters. A member of the ZIP family of divalent metal iron transporters, named ZIPCO, was localised to the plasma membrane and shown to be required for growth of liver stage *P*. *berghei* (Sahu et al., 2014). This growth defect could be rescued by supplementation by iron and zinc, suggesting there is some redundancy in the iron acquisition strategies employed by this parasite (Sahu et al., 2014). However, ZIPCO was only expressed in liver stage parasites, and it remains unclear how other parasite stages acquire iron. ZIPCO is conserved between the apicomplexans, including in *T*. *gondii* where it is predicted to be essential, although has not yet been characterized.

ZIPCO remains the only characterised apicomplexan transporter with a predicted role in iron uptake. However, apicomplexan genomes contain homologues for transporters which have been well characterised in other systems, including the well conserved divalent metal iron transporter _1_ (DMT_1_). DMT_1_ facilitates import of ferrous iron, and other metal ions, into the cell in many systems, including other protozoan parasites (Ballesteros et al., 2018; Smyth et al., 2006), and as such may play a similar role in apicomplexans.

### Intracellular transport and detoxification of iron

The mitochondrion, which requires many heme and Fe-S containing proteins, is the primary destination of iron within the cell. Iron is likely moved into the mitochondrion using the homolog of the yeast mitochondrial iron transporter Mrs3/4, mitoferrin, which is conserved in both *Toxoplasma* and *Plasmodium* genomes although yet to be characterised.

The redox potential of iron which makes it so useful also presents a problem. The reaction of iron with oxygen-containing molecules results in the production of damaging reactive oxygen species (Dixon and Stockwell, 2014). As such the level and distribution of iron within cells must be carefully controlled. Mammals, plants and bacteria use the iron-binding protein ferritin (or similar proteins) to sequester iron in the cytoplasm, however, no ferritin homologs have been found in apicomplexans. Yeast and plants have a different approach to iron storage. Iron is stored in organelles or vacuolar compartments (Li et al., 2001; Sorribes-Dauden et al., 2020; Kim et al., 2006; Roschzttardtz et al., 2009; Zhang et al., 2012). This strategy makes use of membrane transporters to facilitate ferrous iron crossing organelle membranes, likely via a proton-driven antiport mechanism (Kato et al., 2019). Apicomplexan genomes contain homologs for several of these transporters including vacuolar iron transporter _1_ (VIT_1_) (Labarbuta et al., 2017; Sharma et al., 2021; Slavic et al., 2016). PfVIT_1_ is expressed throughout the parasite life cycle and localises to the ER membrane in liver and blood stage parasites. Parasites lacking PfVIT_1_ exhibited reduced parasitaemia and liver stage development, contained more labile iron and were more sensitive to iron stress (Slavic et al., 2016). Interestingly, PfVIT1 appears specific for ferrous iron, while VIT from other organisms are less selective (Sharma et al., 2021; Slavic et al., 2016) suggesting the need for of further metal transporters.

**Figure 1:**
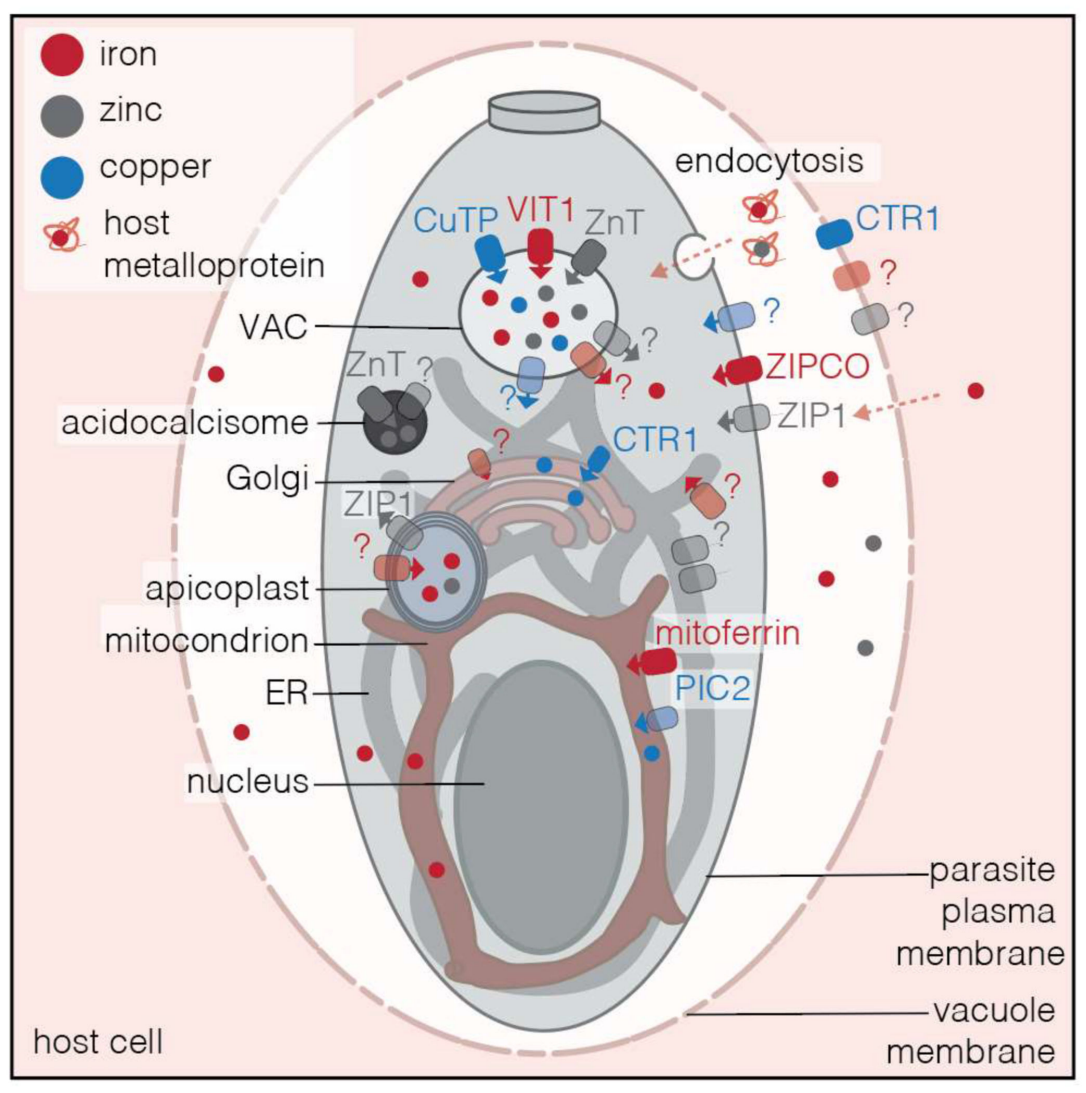
Summary of expected iron, zinc and copper transporters in apicomplexan parasites A schematic showing the major organelles of *T*. *gondii* as a model apicomplexan and the expected localisation of transporters involved in metal transport throughout the cell. As the parasitophorous vacuolar membrane (PVM) is permeable, it is not known if transporters would be required to move metals into the PV space. Apicomplexa appear to be able to endocytose material from the host which may provide a source of metal-containing proteins Iron is required in the mitochondria and apicoplast (see text for details) and may be required in the ER and is likely stored in a vacuolar compartment (VAC). A single apicoplast-localised transporter has been indicated, but it is likely that more than one are required to cross the four membranes of the apicoplast. Zinc is likely required in the mitochondria, ER and Golgi and a zinc transporter has been localised multiple compartments, potentially acidocalcisomes and the vacuolar compartment. Copper is required in the mitochondria and copper transporters have been putatively localised to the VAC and Golgi. Transparent transporters (marked with ?) show the likely location of transporters that have not yet been identified. Iron/iron transporters- red, copper/copper transporters-blue and zinc/zinc transporters - grey.

There remain several open questions as to how iron is moved into other cellular spaces (Fig. 1). Iron transporters into the Golgi and secretory system have been identified in other organisms (Seo et al., 2012; Xiao et al., 2014) and may also exist in the Apicomplexa. Interestingly, the apicoplast contains a dedicated Fe-S biogenesis pathway, essential for organelle maintenance and parasite survival (Charan et al., 2017; Gisselberg et al., 2013). The apicoplast, an essential organelle of secondary endosymbiosis, has long been an attractive therapeutic target for apicomplexan disease. However, despite its requirement for iron, there has been no identification of the transporters required to bring iron across the four membranes of the apicoplast, and as such identification of the mechanism of iron transport would be of particular interest.

### Iron regulation

Whilst the networks which regulate host iron content are well described in other organisms (see (Wang and Pantopoulos, 2011) for an excellent review), regulation of iron uptake and storage in Apicomplexa is not well understood. Mammals regulate iron uptake and storage through aconitase (Alén and Sonenshein, 1999; Marondedze et al., 2016; Tang and Guest, 1999), a dual function enzyme/RNA-binding protein which can interact with stem-loop structures called iron responsive elements (IREs), found in specific mRNAs (Hentze et al., 1987; Koeller et al., 1989). Depending on IRE position, this stabilises or destabilises the mRNA, influencing translation. There is some data to suggest that *Plasmodium* may employ a similar system. *P*. *falciparum* aconitase has been demonstrated to bind both host (Loyevsky et al., 2001) and parasite IREs (Hodges et al., 2005; Loyevsky et al., 2003), however the parasite IREs are highly divergent from their mammalian counterparts and the utility of this system in the Apicomplexa is currently unknown.

While iron is one of the most abundant metals within cells, its uptake, mobility and regulation have not yet been well studied in the Apicomplexa. *Plasmodium* presents an interesting case study, as it faces very different availability of iron throughout its lifecycle, implying the existence of a range of strategies under close regulation.

## Zinc seizure and storage

Zinc is an essential cofactor for a large number of proteins including DNA binding domains, metalloproteases and ribosomal subunits (Cassandri et al., 2017; Eide, 2006). In the apicomplexans, many zinc-binding proteins are conserved, and although the majority have not yet been functionalized, they have been shown to be important throughout the parasites life cycles. For example, zinc finger proteins have been shown to regulate life cycle transitions, and secreted zinc-bound metalloproteases are required for the parasite’s lytic cycle (Gopalakrishnan et al., 2017; Hajagos et al., 2012; Semenovskaya et al., 2020; Tanveer et al., 2013). In *Plasmodium* it has been shown that the parasites accumulate large amounts of zinc (approximately 400% of that found within normal erythrocytes) and that inhibition of zinc acquisition prevents parasite replication (Marvin et al., 2012). A similar, although less dramatic, increase in zinc is seen upon *T*. *gondii* infection (Al-Sandaqchi et al., 2018), highlighting the importance of zinc uptake to parasite replication within their hosts. Interestingly, although zinc appears essential for apicomplexan replication, zinc deficiency or supplementation, either in rodents or in humans, does not appear to alter pathogenesis of *Cryptosporidium* or *Plasmodium* respectively (Hamaguchi et al., 2006; Müller et al., 2001; Veenemans et al., 2011). Zinc sequestration and relocalisation are important facets of nutritional immunity (Vignesh and Deepe, 2016) and it is likely that these successful pathogens have developed highly effective mechanisms for zinc uptake in the face of host efforts.

A number of transporters required for zinc uptake and mobilisation in model organisms have been identified (Eide, 2006). Within mammalian cells, zinc is transported into the cell by a number of high affinity transporters. Proteins of the ZIP (Zrt-, Irt-like Protein) family move zinc into the cytoplasm while cation diffusion facilitator (CDF) proteins move zinc from the cytoplasm to the lumen of membrane-bound compartments. Within a cell, almost all zinc is bound to chaperones which move it to where it is required, e.g. into the ER and Golgi where it can be inserted into newly synthesized proteins. Excess zinc is toxic through a number of mechanisms including by displacing metal cofactors, disrupting protein folding and inducing apoptosis, and so cytosolic levels of free zinc are maintained at a very low level (Maret, 2009; Maret and Krężel, 2007; Plum et al., 2010).

In mammalian and bacterial cells, zinc is removed from the cytosol by zinc efflux transporters, however, in a similar manner to iron, yeast stores zinc within a vacuole (Eide, 2006). Recently the first apicomplexan zinc transporter, named ZnT, was characterised in *T*. *gondii*. ZnT is localised to dynamic, vesicular compartments (Chasen et al., 2019), which, in concert with X-ray microanalysis which localised zinc to suspected acidocalcisomes (Luo et al., 2001; Rohloff et al., 2011) suggests these as the site for zinc storage. Confirming its suspected role, ZnT was found to complement a yeast zinc storage mutant and to be essential for maintaining zinc tolerance within *T*. *gondii* (Chasen et al., 2019). Interestingly, although the ZnT transporter is conserved in *Plasmodium* and highly expressed in late blood stages (Aurrecoechea et al., 2009), the majority of free zinc was observed in the cytosol and mitochondria of blood stage parasites (Marvin et al., 2012) suggesting that zinc storage may be organised differently in *Plasmodium*.

No genes have yet been confirmed to be zinc transporters in *Plasmodium*. However, ZIP_1_, a predicted Zn or Fe permease, was shown to have a role in blood stage replication and was required for gamete production (Kenthirapalan et al., 2016; Sayers et al., 2018). A possible *T*. *gondii* ZIP_1_ homolog (TGME_49__261720) was predicted to localise to the plasma membrane and is essential *in vitro*. CDF, a predicted Zn permease, was also required for male gamete exflagellation and ookinete formation, suggesting a role for zinc in multiple stages of the parasite (Kenthirapalan et al., 2016).

The mechanism of zinc acquisition remains an open question. *T*. *gondii* and *Plasmodium* encode at least 5 other annotated ZIP transporters which could perform this role, however these have not yet been characterised. Further, due to its numerous roles in essential proteins, zinc is likely to be required in other organelles including the mitochondrion, ER and Golgi (Fig. 1). There is currently no direct evidence for a zinc requirement in the apicoplast, although it is possible as the potential zinc transporter ZIP1 contains an apicoplast targeting sequence (Sayers et al., 2018). Mammalian and yeast cells encode transporters to move zinc between organelles (Bafaro et al., 2017; Eide, 2006), however functional homologs of these have not yet been identified in apicomplexan parasites. Given the importance of zinc to cellular functions and the very low levels of free zinc available in mammalian cells (Eide, 2006), how the Apicomplexa acquire zinc is an interesting question for future study.

## Capture and cloistering of Copper

Although only required in very small amounts, copper plays an essential role in cellular processes. It acts as a cofactor in a number of essential enzymes including Zn/Cu superoxide dismutase (Cu/Zn-SOD), cytochrome *c* oxidase and other enzymes involved in diverse pathways such as pigmentation and peptide processing (Balamurugan and Schaffner, 2006). Of these, cytochrome *c* oxidase is conserved in apicomplexans and is essential for energy production. Copper chelation has been shown to prevent *Plasmodium* replication in erythrocytes (Asahi et al., 2014; Rasoloson et al., 2004), although the ability of chelated copper to induce redox stress makes the results challenging to interpret. Copper is also required by *Plasmodium* in the mosquito definitive host (Maya-Maldonado et al., 2021). No studies have yet looked specifically at the copper requirements of *T*. *gondii* or *Cryptosporidium*. However, although copper is likely to be required by *T*. *gondii*, several *Cryptosporidium* species have lost cytochrome *c* oxidase and so may not have a requirement for copper (Liu et al., 2016). Interestingly, even in the absence of cytochrome *c* oxidase, *Cryptosporidium* spp. have maintained at least one copper transporter (a likely homolog of CTR, see below), providing circumstantial evidence for a requirement for copper beyond energy production.

In mammalian and yeast cells, copper uptake occurs through a high affinity copper transporter CTR_1_, copper is then bound to acceptors such as GSH and transported to chaperones (Kaplan and Maryon, 2016). Copper efflux occurs though Golgi-localised P-ATPases which traffic to the plasma membrane upon copper overload, removing copper from the cell (Kaplan and Maryon, 2016). Two putative copper transporters have been investigated in *Plasmodium* (Table 1), a copper transporter CTR_1_ and a CuP-ATPase named CuTP (Choveaux et al., 2012; Kenthirapalan et al., 2014; Rasoloson et al., 2004). PfCTR1 was localised to the erythrocyte membrane and is predicted to be essential during asexual replication in Plasmodium (Choveaux et al., 2012). In *T*. *gondii*, a CTR_1_ homologue is likely essential and is predicted to localise to the Golgi, although no characterisation has yet been carried out. CuTP was localised to vesicular structures within *P*. *berghei* parasite at all life cycle stages and showed some overlap with the vacuolar marker VP_1_ in *T*. *gondii*, suggesting a role of CuTP in copper storage (Kenthirapalan et al., 2014) as the dynamic vacuolar compartment is also associated with zinc storage (Chasen et al., 2019). CuTP was not shown to be essential for blood stage growth or gametocyte production in *P*. *berghei*, however is essential in gametocyte fertility (Kenthirapalan et al., 2014), suggesting that this life cycle stage has an altered requirement for copper. The Apicomplexa also encode a homolog of the mitochondrial phosphate/copper transporter PIC_2_ (Zhu et al., 2021) which is predicted to be essential and mitochondrially-localised in *T*. *gondii*, however has not yet been characterised.

The effects of *Plasmodium* infection on host cell copper are also unclear, previously it was shown copper levels either decrease (Rasoloson et al., 2004) or increase (Marvin et al., 2012) in trophozoite-infected red blood cells. While *T*. *gondii* infection appeared to increase the copper content (Al-Sandaqchi et al., 2018). Within cells, very little free copper is present with the large majority bound to host cell proteins making copper acquisition a challenge for intracellular pathogens (Li et al., 2019).

In summary, the role of copper in apicomplexan development deserves further investigation. There is evidence that maintenance of copper homeostasis is required by the parasites, and some of the genes involved in this process have been identified and initially characterised (Table 1 and Fig. 1). However, the topic merits systematic investigation as copper homeostasis has been shown to be vital in pathogenesis of a number of pathogens (Li et al., 2019).

## Summary

Despite the importance of Apicomplexa, both clinically and in veterinary practice, the study of the uptake, use and storage of transition metals lags behind work done in comparable pathogens. Transporter characterisation has improved in recent years, and the identification of several metal transporters has underlined the importance of metals to the parasites, however a number of predicted transporters remain unknown. Further, the role of transcriptional and post-transcriptional regulation in metal uptake and storage has not been addressed, despite these processes being tightly controlled in other systems. This limits our ability to understand how these pathogens interact with their hosts, and how they respond to and overcome host nutritional immunity.

As highly divergent parasitic eukaryotes which often cycle between mammalian and insect hosts, there may well be important biological differences in how metals are handled which could be exploited therapeutically. One interesting area for future investigation is the apicoplast, which depends on iron import (Gisselberg et al., 2013), but has no identified iron transporters. By identifying the strategies employed to transport metals around the cell we have the opportunity to learn more about how the common problem of metal acquisition and storage has been addressed across the broader tree of life.

## Figures and Tables

**Table 1:** Genes involved in copper homeostasis in apicomplexan parasites. P. falciparum phenotype data from (Zhang et al., 2018). T. gondii phenotype data from (Sidik et al., 2016), values > ~ -1.5 are considered dispensable, LOPIT data from (Barylyuk et al., 2020).

Plasmodium					T. gondii		
Type	Gene name	Pf ID	Phenotype	Localisation	Tg ID	Phenotype score	Localisation prediction
Transporter	CTR_1_	PF_3_D_7_1439000_	Essential	Translocates from the erythrocyte plasma membrane in early ring stage to a parasite membrane as the parasites developed to schizonts (Choveaux et al., 2012)	TGME_49_262710_	-2.62	Golgi
Channel	CTR_2_	PF_3_D_7_1421900_	Essential	-	TGME_49_249200_	2.31	-
ATPase	CuTP	PF_3_D_7_0904900_	Dispensable	Expressed in all Plasmodium life cycle stages. Localizes to vesicle-like structures (Kenthirapalan et al., 2014)	TGME_49_201150_	-1.57	Endomembrane vesicles,colocalises with the VAC (Kenthirapalan et al., 2016)
Metallochaperone	Cox17	PF_3_D_7_1025600_	?	Cytoplasmic localisation in asexuals (Choveaux et al., 2015)	TGME_49_240550_	-2.7	mitochondrial
Mitochondrial copper/phosphate transporter	PIC_2_	PF_3_D_7_1202200_	Dispensable	-	TGME_49_278990_	-2.7	mitochondrial
